# Enhancing IoT Security: An Innovative Key Management System for Lightweight Block Ciphers

**DOI:** 10.3390/s23187678

**Published:** 2023-09-05

**Authors:** Muhammad Rana, Quazi Mamun, Rafiqul Islam

**Affiliations:** School of Computing, Mathematics and Engineering, Charles Sturt University, Wagga Wagga, NSW 2678, Australia; qmamun@csu.edu.au (Q.M.); mislam@csu.edu.au (R.I.)

**Keywords:** key management, pre-distribution, partial key, lightweight cryptography, block cipher, Internet of Things (IoT), security

## Abstract

This research paper presents a study on designing and implementing a robust key management scheme for lightweight block ciphers in Internet of Things (IoT) networks. Key management is a critical concern for IoT devices due to their limited resources and susceptibility to security threats. The proposed scheme utilises partial key pre-distribution to achieve lightweight and secure key management. The protocol’s security has been analysed against various attacks, demonstrating its resistance. Performance evaluation results indicate that the proposed key management technique is suitable for resource-constraint IoT networks, as it reduces communication overhead, power consumption, and storage space requirements. The methodology employed in this research includes designing and implementing the proposed key management scheme and conducting scenario-based analyses of its functionality. The results affirm that the proposed solution effectively ensures secure communication in IoT networks. Overall, this research contributes to developing a secure and efficient key management scheme for lightweight block ciphers in IoT networks.

## 1. Introduction

The rapid expansion of the Internet of Things (IoT) is projected to connect 75 billion devices by 2025, resulting in a vast amount of generated data [1]. Consequently, ensuring the security of IoT networks becomes increasingly crucial. IoT security strategies prioritise maintaining information accuracy, confidentiality, and accountability. To contribute to the evolving IoT security paradigm, our research focuses on developing a lightweight cryptographic protocol capable of establishing within resource-constrained IoT environments [2,3,4]. This project involves designing and implementing various components of cryptographic protocols, including S-boxes, P-boxes, encryption, and decryption techniques, to create a robust block cipher-based solution. This paper proposes a key management scheme as an integral part of our IoT security project.

In any cryptographic system, effective key management is essential for maintaining the confidentiality and integrity of transmitted data. Proper management of security keys significantly reduces the risk of unauthorised data access [5,6]. Moreover, in an IoT environment, where secure communication among IoT devices is paramount, key management plays a pivotal role in safeguarding data transmission and protecting against potential cyber-attacks [7].

The security and privacy of resource-constraint IoT environments present several challenges. The security methods employed are susceptible to a range of known and unknown attacks because of their multiple security weaknesses. Some of these methods also suffer from inefficiencies related to computational and transmission overhead [8]. Hence, developing an efficient key management strategy for lightweight block ciphers in the context of a resource-constraint IoT environment is imperative. This study proposes a robust key management scheme in response to this requirement.

The key management process encompasses several steps: key generation, storage, protection, transfer, loading, utilisation, and destruction. Key analysis determines the number of keys required for the network and individual nodes and the keys that need updating for enhanced security. The key assignment involves a cryptographic procedure where a trusted entity generates user secrets and converts them into user keys, requiring agreement among all parties involved. The generation of communication keys by nodes follows, requiring trust in the key-generating nodes. Key distribution occurs after network deployment, utilising logical topologies like cluster-based organisation for IoT devices.

We identify four stages, (1) key analysis, (2) key assignment, (3) key generation, (4) key distribution, and the following approach while designing the proposed key management scheme for a resource-constrained IoT environment. We limited the number of message passings between the IoT devices for key generation. For key storage, transfer, and utilisation, we considered reducing the key sizes to maintain the security levels but reduce the total space requirement. We used a concatenation algorithm for key assignments and updates requiring low processing power. Key revocation and update procedures are implemented, along with authentication and authorisation mechanisms. Efficiency optimisation was pursued by minimising communication overhead and computational complexity. The scheme undergoes security analysis, prototyping, and testing, with evaluation and iteration to refine its effectiveness. This approach ensures a tailored key management scheme that addresses resource limitations while upholding security, efficiency, and compatibility with IoT devices and systems. The key contributions of this paper include the following:

- Designing a lightweight key management scheme for block ciphers in IoT environments;
- Utilising partial keys to increase resilience;
- Implementing pre-distribution methods to reduce key exchange-related messages between nodes, conserving space and energy in the resource-constrained IoT environment.

We aim to address IoT networks’ specific security challenges by designing a comprehensive key management scheme. The proposed scheme seeks to ensure secure data transmission, protect sensitive information, and mitigate the risks associated with unauthorised access. This research contributes to the broader objective of enhancing IoT security and aligning cryptographic solutions with the unique characteristics and limitations of resource-constraint IoT environments. Through our proposed lightweight cryptographic protocol and key management scheme, we aim to establish a robust security foundation for the expanding IoT landscape, fostering secure communication and safeguarding the integrity of transmitted data.

## 2. Related Work

Existing key management schemes for IoT can be categorised based on various factors, including the location and control of key management functions (centralised vs. distributed), the type of cryptographic algorithms used (symmetric vs. asymmetric), the establishment of keys (pre-shared vs. dynamic), the assignment of keys (group vs. individual), the structure of the scheme (hierarchical vs. flat), the trust model employed, and the specific application focus. Considering these categorisations makes it easier to understand and compare the different key management schemes available for IoT, enabling the selection of the most suitable scheme based on the specific requirements and constraints of the IoT deployment. Below, we briefly describe some existing key management schemes.

Symmetric cryptography uses duplicate keys between the sender and the receiver. For n entities to securely communicate, K=n∗(n−1)/2  keys are needed because every pair of communicating nodes shares a secret key. The Symmetric Threshold Multipath (STM) approach combines symmetric key management with secure online key distribution. The unique strength of this method lies in its utilisation of symmetric key distribution. An extra layer of security is introduced by employing a threshold secret-sharing mechanism to divide the symmetric key for each message [9]. Including multipath techniques and a pre-distributed symmetric key management scheme further enhances the protection of secret share transfers. However, it is essential to note that the IoT environment may pose challenges for online key distribution due to increased processing power requirements [10]. The Compression–Encryption Model (CEM) consists of dynamic key creation, chaos-based selective compression, and symmetric key distribution stages. In the key distribution model, the End of File (EoF) approach embeds the authenticated, encrypted key into the cipher image. The generated symmetric keys undergo a run test to demonstrate randomness and dynamism. Visual and statistical analysis confirms the proposed model’s resistance against various attacks.

Sourabh Chandra et al. [11] introduced a secure data encryption method called Caesar Cipher symmetric key encryption. This approach allows the encryption of plaintext containing integers, special symbols, and case-sensitive characters. The suggested method utilises normalised encryption with the Caesar Cipher key. It is important to note that the technique works best with special characters and numbers in the final cipher text, which may limit its usability for broad use. Priyadarshini Patil et al. [12] propose a secure cipher text generation method using random substitution combined with double-column transposition ciphers in their research paper. The generated key is partitioned into two keys for enhanced security. Even with 256 key-based cryptanalysis attempts, brute-force decryption of the cipher is unfeasible. However, it is crucial to consider that this method utilises larger keys, which may pose challenges for IoT end devices with limited capabilities.

The transmitting node encrypts with the recipient’s public key, which all nodes can see, and the receiving node decrypts with his hidden private key. Asymmetric key cryptography uses fewer keys than symmetric. For n communicating nodes, K=2∗n  keys. We list asymmetric key cryptography algorithms here. Asymmetric Lightweight Key Management System (ALMS) introduces Vehicular Ad Hoc Networks (VANET) group key management. ALMS efficiently integrates the Chinese Remainder Theorem (CRT), prime factorisation, discrete logarithm, and noise parameters to create a centralised group key management mechanism. The suggested protocol overcomes numerous key management protocol issues, including high computational and communication costs. Performance, complexity, and security analyses prove ALMS works [13]. This asymmetric key management protocol is tailored explicitly for Vehicular Ad Hoc Networks (VANETs).

Zhang et al. [14] introduced a matrix-based cross-layer key establishment protocol for smart homes, addressing the heterogeneity of domestic devices. The protocol utilises a master key and session keys, with the master key shared between devices and the cluster head. The protocol achieves low energy consumption by delegating heavy operations to the powerful home gateway, enabling key refresh and network scalability. It offers low storage costs for appliances but requires 2λ+1 multiplication computations for session key establishment. It has higher communication costs than other protocols, where λ represents the total number of nodes. Nafi et al. [15] present a lightweight matrix-based key management protocol for IoT networks that decreases communication and storage overheads at constrained nodes. Nafi’s approach includes initialisation, pairwise and group key setup, new node addition, key revocation, and key renewal stages. An identification number (Idi), a square matrix M of order n, and a one-way keyed-hash function. Hash (msg, k) are stored in the node’s memories in the pre-deployment phase. On the other hand, matrix-based pairwise key management demands increased computational power.

Messai and Seba [16] introduced EAHKM+, a new key management scheme for hierarchical wireless sensor networks. It consists of key pre-distribution and cluster formation with the key establishment. The scheme pre-assigns three keys to each sensor node, a network key and two pairwise keys, and deletes the network key after the second phase. Secure clusters are formed, and new keys are established. EAHKM+ enables flexible cluster management and supports a large number of nodes. However, the rekeying process is computationally and communication-wise costly due to the need for rerunning the clustering algorithm.

S. Mesmoudi et al. [17] recently proposed a dynamic key management scheme for hierarchical wireless sensor networks called SKWN. It incorporates key establishment, key renewal, and new node addition, using machine learning to adapt the security level based on network activity. The scheme utilises an Intelligent Security Agent (ISA) component to detect and address intrusions in real time, offering scalable, flexible, and reliable security. However, the scheme involves a relatively high number and size of exchanged messages during different phases. Soumyashree et al. [18] describe a blockchain-based distributed IoT architecture with secure key management. The proposed architecture leverages blockchain technology’s openness, traceability, and fault tolerance to enhance data privacy protection in IoT scenarios and establish a secure environment for interaction. The project uses a one-way hash chain to give each IoT device public and private keys that may be used to check the other pair. Experiments demonstrate that the proposed strategy works better than average. Using public and private keys in this approach necessitates more storage and processing power for IoT.

Based on the related work described above, we identify the following research gaps that need to be addressed:

1. Limited consideration of resource constraints: While some schemes address the resource constraints in IoT devices, such as low processing power and limited storage, there is a need for more comprehensive approaches that specifically focus on designing lightweight key management schemes tailored to the constraints of IoT devices.
2. Scalability and efficiency: Several existing schemes demonstrate effectiveness in specific scenarios or network configurations, but scalability and efficiency are crucial factors for large-scale IoT deployments. A significant research gap is apparent in the field of key management schemes designed for the evolving IoT landscape, characterised by an exponential growth in the number of IoT devices. These schemes need to ensure both efficient handling of a rapidly increasing number of IoT devices and the maintenance of secure communication protocols.
3. Adaptability to dynamic environments: IoT environments are dynamic, with devices frequently joining and leaving the network. Existing schemes often lack mechanisms to handle the dynamic nature of IoT networks, such as efficient key renewal, new node addition, and key revocation processes. Research is needed to develop key management schemes that adapt effectively to the changing IoT network topology.
4. Consideration of diverse IoT applications: The related work primarily focuses on generic IoT scenarios, but different IoT applications may have specific security requirements and constraints. Future research should explore key management schemes tailored to particular IoT applications, such as smart homes, healthcare, or industrial IoT, considering their unique characteristics and security needs.

Addressing these research gaps will contribute to developing more effective and efficient key management schemes designed explicitly for resource-constrained IoT environments, enabling secure communication while considering the limitations of IoT devices. Our proposed scheme includes pre-distribution of secret partial key, symmetric encryption, and decryption, changing secret keys in each round without communicating with the participating IoT devices, authentication and authorisation by the cluster head, and consideration of diverse IoT applications. Below, we mention the contributory factors of the proposed approach, and we will discuss them elaboratively in Section 8:

- Simplified key establishment;
- Reduced communication overhead;
- Lower computational requirements;
- Enhanced security;
- Scalability;
- Resistance against key-related attacks;
- Adaptability and flexibility.

## 3. Cryptographic Key Management

In the Introduction section, we mentioned the four key tasks for a key management scheme. In this section, we describe them elaborately. This discussion is required as our design approach will be tailored based on this discussion.

Key management comprises four main tasks: analysing, assigning, making, and distributing keys. Keying needs are examined during key analysis to identify the number of keys required for the network and the number of keys needed for each node. Additionally, a study may be performed to determine the keys that require an update. The proposed approach performs better on resource-constrained devices. Decentralised architecture improves scalability and decreases device overhead.

### 3.1. Analysis of Key

The number of bits in a key that are utilised in cryptographic encryption procedures is referred to as key length or key size. The method’s complexity determines the encryption strength, which prevents the key from being discovered. The key size used in the encryption process determines the encryption intensity. A longer key length assists the algorithm in generating greater complexity, which improves network communication security. However, such a long key requires more processing power and memory, which IoT devices may not have.

On the other hand, a shorter key length increases the chances of being hacked by attackers [2]. AES, LED, RECTANGLE, and PRINCE use 128-bit keys, making them inappropriate for systems with limited resources [19]. However, the Modified QARMA method uses just 64-bit keys for encryption. Due to their 80-bit key size, SAT Jo, Modified PRESENT, Piccolo, and KTANTAN are suited for IoT devices [3]. However, the updated PRESENT cipher must undergo additional power consumption testing before being considered [20]. The trade-off between security and key length must be addressed to design efficient encryption for resource-constrained edge devices [2].

It is important to remember that while creating a new block cipher, especially one with resource-constrained natures, we are searching for more than just creative ways to implement it. Instead, the cipher’s design and implementation are linked, revealing some intrinsic limitations and conflicts previously overlooked. For instance, a specified security level establishes minimum block and key lengths. Simply processing a 64-bit state with a 128-bit key imposes fundamentally reduced space requirements. Furthermore, we found that hardware implementation, especially small hardware implementation, favours recurrence [21]. In our cryptographic protocol, the deliberate use of a 64-bit key size stems from a careful balancing act between various factors. While it is commonly known that larger key sizes contribute to better protocol security, we must consider the associated computation and processing time costs.

Our decision to settle on a 64-bit key size was driven by the necessity to find an ideal balance between security and efficiency. We wanted to ensure that the computational demands and processing time remained reasonable and that the protocol could be implemented without excessive overhead. By choosing a 64-bit key size, we aimed to avoid the potential downsides of very large key sizes, such as increased computational complexity. It is worth noting that lightweight cryptographic protocols such as LED and PRESENT use 64-bit and 80-bit keys, respectively [19].

One key factor that guided our choice was the dynamic nature of our protocol. We frequently change the keys for each round of communication, meaning that the keys have a very short lifespan compared to other existing protocols. This unique feature strongly influenced our decision to select a 64-bit key size. Given the keys’ transient nature and frequent renewal, we found that a 64-bit size struck the right balance between maintaining strong security and keeping computational demands manageable for resource-constraint IoT devices.

In this research, we propose a partial key (half key) rather than a full key to reduce overhead and make the cipher more robust in secure communication. Using partial keys has two advantages: (i) less storage is required, and (ii) an attacker cannot access the encryption/decryption keys if the IoT device is taken. Two neighbouring devices establish their encryption/decryption key by adding their partial keys.

### 3.2. Assignment of Key

The process of assigning keys to different nodes is known as key assignment. A key assignment approach is a cryptographic procedure that enables a trustworthy party (e.g., the data owner) to produce user secrets. It also provides a key generation mechanism that allows users to derive their corresponding keys from their provided secret. When establishing a secure communication channel, the participants in that channel’s establishment agree on the keys that will be used to communicate. Key allocation can be static or dynamic, depending on the key management method.

The proposed key management has two basic characteristics: (i) the pre-distribution of keys and (ii) the use of partial (half) keys rather than full keys. There were several reasons why we used pre-distribution of keys rather than assigning keys dynamically for our cryptographic protocol in resource-constrained IoT devices.

*Resource efficiency:* Resource-constrained IoT devices often have limited computational power, memory, and energy resources. Pre-distributing keys eliminate the need for complex key exchange protocols or computationally intensive cryptographic operations during runtime, which helps conserve resources and improve device efficiency.

*Lower communication overhead:* Dynamic key assignment typically involves exchanging messages between devices or with a central authority to establish and update keys. This process may introduce additional communication overhead, consuming valuable bandwidth and increasing latency [22]. Pre-distribution of keys eliminates the need for frequent key exchanges, reducing communication overhead [23].

*Security benefits:* Pre-distribution of keys allows for stronger security guarantees. With dynamic key assignments, intercepted or compromised keys are always risky during the exchange process. In contrast, pre-distributed keys can be securely provisioned during the device manufacturing phase, reducing the potential vulnerabilities associated with key exchange protocols.

*Scalability:* Pre-distribution involves provisioning devices with keys prior to their deployment or activation, establishing a foundation of secure communication from the outset. This approach streamlines the process of onboarding new devices, eliminating the need for real-time key exchanges and reducing potential bottlenecks as the network expands. Bechkit et al. [24] show that key pre-distribution can help to achieve high scalability for sensor networks.

In our approach, even if the same partial keys are used across all nodes, the resultant full key remains unique for each communication. This uniqueness is achieved as each node creates its own order list, which is then shared among the nodes. During every communication round, nodes employ the shared and their own order lists to generate a new full key. This step ensures that the final full keys are distinct and secure even with a common set of partial keys. We recognise the importance of emphasising this dynamic in our study and provide a comprehensive explanation to address the concerns.

*Offline operation:* Resource-constrained IoT devices may operate in disconnected or intermittent connectivity environments. Pre-distributed keys enable these devices to operate securely even without constant network connectivity. Since the keys are already available on the device, cryptographic operations can be performed offline without the need for additional communication.

*Resistance to certain attacks:* Pre-distributed keys can provide resilience against certain types of attacks, such as man-in-the-middle attacks during key exchange. While a secure key exchange with appropriate authentication can reduce the likelihood of man-in-the-middle attacks, it can also introduce additional overhead to the resource-limited IoT environment. By eliminating the need for dynamic key assignment, the protocol can be designed to mitigate specific vulnerabilities associated with key exchange protocols.

Pre-distribution involves adding a key pool to each IoT device at the time of deployment. The pre-distribution of keys is widely regarded as secure, just as the IoT system is secure during deployment. Pre-distribution is more suitable for nodes with limited resources, such as IoT sensor nodes, because it reduces the communications required to distribute the pool keys. Additionally, pre-distribution can help identify intruders if an external IoT device or node attempts to utilise a non-listed key during the pre-distribution stage.

### 3.3. Generation of Key

The process of making cryptographic keys is known as key generation. Two types of keys are generated—communication keys and administrative keys. To establish a communication key, nodes exchange messages and then apply pre-agreed functions to create communication keys. For instance, in the proposed scheme, two nodes concatenate partial keys to generate encryption/decryption keys. On the other hand, administrative keys can be produced once or repeatedly during a network’s lifetime.

In the proposed key management scheme, when two nodes in a cluster need to establish communication, they generate their encryption and decryption keys by applying a concatenation function to their partial keys. The concatenation function is chosen for its simplicity. When the partial keys are selected from a pool of keys and assigned to each node, each node selects the order in which its partial keys will be used and sends this order list to the other node with which it wishes to communicate. All the nodes are authenticated using the administrative keys before they participate in establishing communication keys. Combining their partial keys according to the agreed order allows the two nodes to generate full keys using a predefined function.

**Figure 1:**
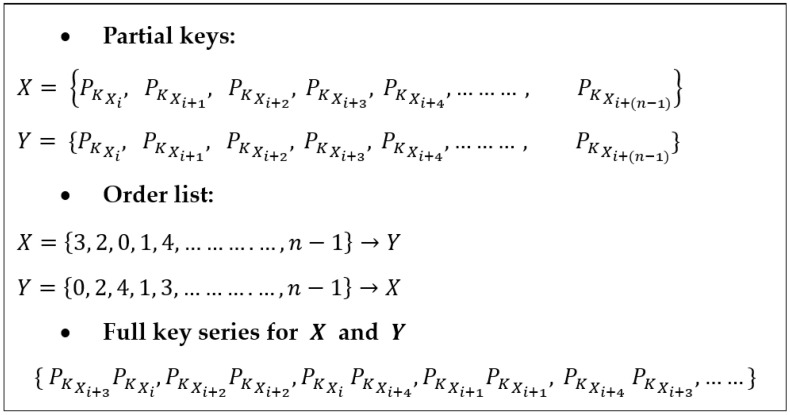
Key generation from the order list.

This approach allows a node to generate multiple encryption and decryption keys with a single message passing. In each communication round, the two nodes use a unique key to ensure the data’s freshness and enhance IoT’s resilience against attacks. Figure 1 illustrates the generation of the full key from the order list.

To better understand how this scheme works, let us consider an example where nodes X and Y have been assigned a list of partial keys {a, b, c, d, e, f}. Node X sends its order list {3, 2, 0, 1, 4} to node Y, while node Y sends its order list {0, 2, 4, 1, 3} to node X. These order lists are randomly created, ensuring independence between them, although they have the same cardinality. Based on the received order lists, node Y knows that the partial keys selected by node X, in their respective order, are {d, c, a, b,e}. Similarly, node X knows that the partial keys selected by node Y, in their respective order, are {a, c, e, b, d}.

Once nodes X and Y agree on a concatenation function to build the full key from their partial keys, they both know that the resulting full key series will be {da, cc, ae, bb, ed}. Consequently, in the first round of message passing, both X and Y will use “da” as the encryption and decryption key; in the second round, they will use “cc”, and so on.

The cluster heads in the proposed key distribution scheme contain the universal set of partial keys. Each cluster head then assigns the local set of partial keys, which all nodes in the respective cluster keep. All nodes are assigned the universal set of partial keys during the pre-distribution phase. After receiving the local set of partial keys from its cluster head, a node deletes the partial keys from the universal set that are not in the local set. As a result, the requirement of knowing the number of partial keys remains linear all the time. When a node in cluster A needs to communicate with a node in Cluster B, they communicate via the cluster head. There, nodes in different clusters do not need to know the local sets of each other.

This methodology enables efficient key generation and management, ensuring secure communication between resource-constrained IoT devices while maintaining the freshness of the keys for each communication round.

### 3.4. Distribution of Key

A reliable key distribution system is required so that nodes can safely communicate with each other. Key distribution is the process of delivering keys to their designated nodes after they have been generated and assigned to them. Communication keys are often shared after the network has been implemented. Communication keys are used for a brief duration and should be refreshed frequently, which may involve analysis, assignment, generation, and redistribution. IoT nodes are low-power devices with limited processing power, memory, communication bandwidth, and energy. Due to their limited power and resource capacities, IoT nodes are vulnerable to data overload challenges [25]. The main goal of the security protocol is to reliably protect the confidentiality and authenticity of data transmitted to and from IoT nodes [26]. This proposed key management technique does not involve any further transmission of keys over the channel beyond the initial pre-distribution phase. Sending only an updated order list through the encrypted channel eliminates the need to send any partial keys. As a result, IoT devices can save communication costs without sacrificing security by exposing keys during transmission.

## 4. Challenges and Issues in IoT

The Internet of Things (IoT) presents several challenges and issues that have an impact on the design of the key management scheme, including resource constraints, a variety of device kinds, a high vulnerability to security breaches, unplanned communications, the ability to understand its surroundings, and the absence of supporting infrastructure.

*Lacking sufficient resources:* IoT devices have limited connectivity, memory, and processing power [27]. Unlike older RFID tags with limited processing capabilities, contemporary RFID devices are semi-passive and allow in-device processing [28]. Backscattering, energy harvesting, and wireless power transmission are proposed to power resource-constrained IoT devices [29,30,31]. Ambient backscatter allows battery-free tags to transmit data and harvest energy from wireless signals. A similar distributed backscatter protocol was presented by Ma et al. [32] for use in large-scale IoT networks. Energy harvesting in a Wi-Fi-based IoT network that links IoT devices for power transfer and data transmission was studied by Khairy et al. [33].

The limited resources of IoT devices can significantly impact key management schemes. Since IoT devices often have restricted memory and processing capabilities, complex key management algorithms may be impractical. This vulnerability can result in a compromise between security and resource constraints. Furthermore, the limited power of IoT devices means that frequent key updates and cryptographic operations can quickly deplete their battery life. As a result, key management schemes for IoT devices must be designed with these resource limitations in mind, such as using lightweight cryptographic algorithms and optimising key update intervals to balance security and energy consumption.

*Diversified hardware:* Sensor technology and embedded IoT devices enable low-cost computer platforms. Low-power radios minimise IoT energy dissipation and impact. IoT uses high-order computational devices, sensors, and embedded devices to switch, route, and process data [34]. The diversified hardware in IoT devices can impact key management schemes by making it difficult to develop a standardised approach to key distribution, storage, and encryption that can be implemented across all devices. Diversified hardware can result in compatibility issues, affecting the system’s overall security. Additionally, the varying hardware capabilities can make it challenging to implement specific security protocols, such as those that require extensive computational resources, on specific IoT devices.

*Attack vectors in security:* Security threats and attack vectors are prevalent in various applications of Internet of Things (IoT) network environments. Due to its ubiquity and connectivity, the IoT is vulnerable to attacks on multiple networks and applications. This vulnerability of attack makes it hard to build a security framework for the IoT that is consistent, scalable, and works with other systems [35]. Security attack vectors can impact a key management scheme by exploiting key distribution, key compromises, and encryption vulnerabilities. These vulnerabilities can result in unauthorised access and data breaches, compromising the security and integrity of the system. Addressing potential attack vectors in the key management scheme is critical to ensuring system security.

*Unplanned communication:* An unforeseen interaction is expected in IoT systems, where an object may unexpectedly move in and out of its communication range, leading to an unanticipated interaction. For instance, an event can be automatically generated when a user’s smartphone comes into contact with a home appliance like an air conditioner, freezer, or CCTV. Such interactions between objects in the IoT network can transmit events into the network [36]. Unplanned communication in IoT systems can impact the design of a key management scheme by increasing the risk of unauthorised access to sensitive data transmitted by the devices. An unexpected interaction between objects can potentially compromise the system’s security, resulting in the loss or theft of keys used for encryption. Therefore, it is essential to consider the potential impact of unplanned communication on the key management scheme and implement robust encryption algorithms, secure key distribution, and storage mechanisms to mitigate the risk of security breaches.

*Location and context awareness:* Location and context awareness are crucial factors in the IoT domain. A significant quantity of sensors produces substantial volumes of data, which lacks significance until it undergoes analysis and interpretation. Context awareness significantly enhances data interpretation and the self-governing, flexible conduct of Internet of Things (IoT) devices. The impact of location on IoT interactions renders it a critical element of context-aware computing [37]. Location and context awareness can impact the design of a lightweight key management scheme by providing contextual information that can be used to implement more efficient and secure key distribution and storage mechanisms. For instance, contextual information such as device location and user behaviour patterns can be used to adjust encryption settings and key distribution strategies to optimise energy consumption and enhance security. Incorporating location and context awareness into the key management scheme can lead to more efficient and effective management of cryptographic keys in IoT devices.

*A dynamic network lacking infrastructure:* Wireless, battery-powered, and resource-constrained IoT devices can easily join and leave the network, resulting in dynamic nodes. Nodes in an IoT network can become dynamic due to various reasons, such as depleted battery life or poor wireless connections. Maintaining a stable IoT network poses a significant challenge in locations with inadequate fixed infrastructure. Interaction between nodes is essential to ensure the network’s functionality and connectivity [38]. A dynamic IoT network lacking infrastructure can impact the design of a lightweight key management scheme by requiring a decentralised and flexible approach to key distribution and management. In such scenarios, it is necessary to have key management mechanisms that can handle the dynamic entry and exit of nodes in the network while ensuring secure communication between them. Lightweight cryptographic algorithms and efficient key storage mechanisms are essential to conserve limited resources and optimise energy consumption.

## 5. Key Management in Cluster-Based IoT Network

End IoT devices often use a topology based on clusters, as discussed in [39]. The base station (BS) receives data from the end nodes, processes it, and provides the data to the end user. It is usually a stationary device far from the IoT end nodes. BS do not have limited resources like IoT end nodes, so they can process and store a lot of information. One node in each cluster is chosen to be the cluster head (CH). The IoT devices and the BS talk to each other through the cluster head, which acts as an interface. The CH’s job is to carry out common tasks for the entire cluster, such as giving instructions to the nodes that make up the cluster and consolidating data before it is sent to the backend server. As a result, the cluster nodes go into the CH, and the cluster heads go into the base station. Messages sent and received by a member node in a cluster-based topology are limited to the cluster head CH. To transmit data to the base station, cluster heads from various clusters may communicate with each other.

Overhead concerns make public-key cryptography a pricey option for securing IoT end networks [40]. Most end IoT devices do not have enough memory to store large public keys. Also, IoT end nodes require much time and energy to compute public keys as they have limited processing power. As a result, symmetric key management has been chosen to be applied to the suggested key management strategy. The term symmetric cryptography can be described as follows: $\mu^{\prime }=E_{k}\left(\mu \right)$ is a message that has been encrypted, where k denotes the secret key, μ denotes the plain text, and E denotes the encryption algorithm. Therefore, the decryption of the identical message is represented by the equation $\mu =E_{k}^{1}\left(\mu \right).$

This study proposes a key management system based on symmetric cryptography and partial key pre-distribution. Due to the resource constraints nature of IoT devices, symmetric cryptography and pre-distribution of keys are both suitable for IoT architecture. Instead of storing the complete set of keys, each IoT device would record a set of partial keys. This provides the benefit of requiring less storage space while also ensuring that an attacker cannot access the encryption/decryption keys even if an IoT device is captured.

Concatenating partial keys creates an encryption/decryption key for two communicating devices in a cluster. To keep the key management system safe, IoT nodes in a cluster can create a vast number of keys, and each pair of IoT nodes uses a unique communication key. The encryption and decryption of data between the two communicating nodes are further enhanced by employing a new secret key in each round. IoTs can achieve data freshness and resilience to assaults without creating a long nonce through this feature.

## 6. Various Communication Links in IoT Networks

**Figure 2:**
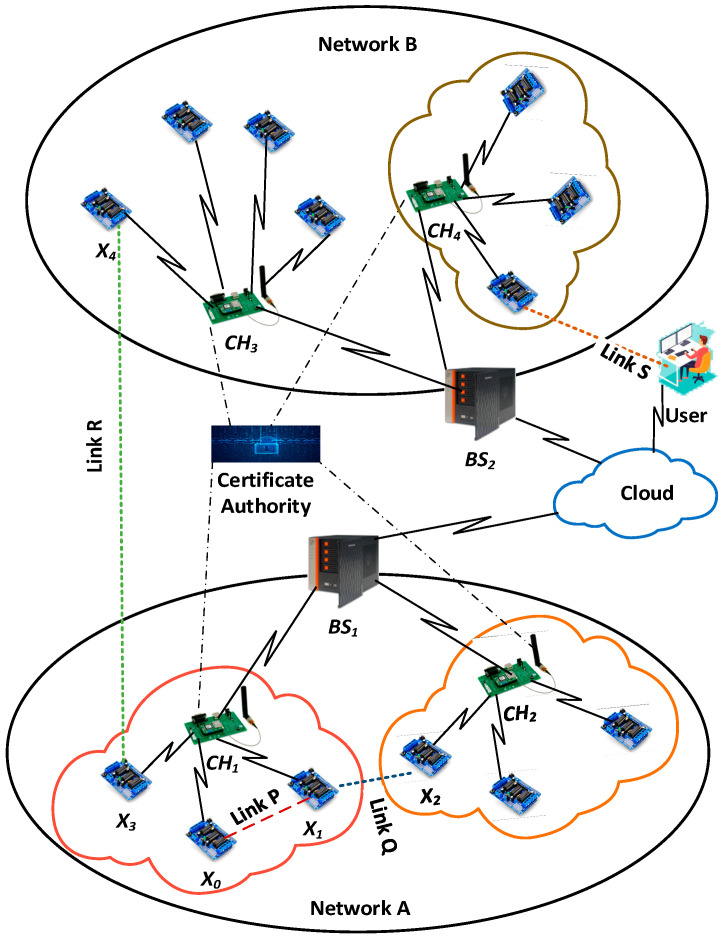
Various communication links (link P, Q, R).

These cluster heads, base stations, end-users, and IoT nodes should be able to securely communicate in an IoT environment to gather information from end devices in a cluster-based IoT environment. Four distinct forms of network connectivity are depicted in Figure 2 for cluster-based systems.

1. Communication between two IoT devices within the same cluster (link P);
2. Communication between IoT nodes in multiple clusters within the same network (link Q);
3. Communication between two IoT nodes in different clusters and on various networks (link R);
4. Communication between an IoT node and an end user (link S).

IoT networks consider two classes of network devices: devices with many resources, like cluster heads and base stations, and network devices with limited resources, like IoT end devices. All communications in a specific cluster are routed through its cluster head in this cluster-based IoT architecture. The cluster head is considered the certificate authority (CA) for the same group of IoT nodes in the group of devices, which is like giving out implicit certificates. Edge nodes are expected to know the cluster head during the pre-distribution phase. Our principal concerns are the secure communications between constrained nodes or between a constrained node and a resource-rich entity such as a CH.

All cluster heads and end-users should use Datagram Transport Layer Security (DTLS) for secure communication after acquiring X.509 certificates issued by a common certificate of authority, primarily managed by end-users and cluster heads due to their resource capability. Over an Internet of Things cloud channel, resource-rich network entities first communicate with the common CA [41]. Suppose an end-user wants to talk to an IoT node in a particular cluster. In that case, it must first set up a secure Datagram Transport Layer Security connection with the cluster head and obtain implicit certificates from the corresponding cluster head. The end-user can get the certificate from the CH by using this secure link. Finally, the end-user can use the certificate to talk to the IoT node. As the security of resource-restricted environments is our primary research emphasis, this paper does not provide additional information regarding the authentication between two resource-rich devices, such as a base station, cluster head, and an end-user.

Adding a new device to the existing network is straightforward, regardless of the network size. When both parties possess legitimate implicit certificates, they can verify each other even if they are on different local networks. Existing nodes can dynamically change their locations once they have requested a fresh certificate. A cluster head is considered capable of recognising legitimate devices and communicating with network entities seeking security credentials. The cluster head must validate the identification of the certificate requestor at the initial communication. It does so primarily based on the identity of the requestor node. Medial Access Control (MAC) addresses are considered while attempting to identify a device. This study proposes an end-to-end verification method for the application layer. The application layer is safeguarded using IEEE802.15.4 security techniques from the physical and MAC levels [42]. After validating each other and creating secure communication links, the distributed nature of the architecture enables edge nodes, CHs, BSs, and end-users to verify each other.

## 7. The Proposed Key Management Scheme

The suggested key management method is explained using several notations. A list of such notations is shown in Table 1. Table 2, Table 3 and Table 4 show key management between different nodes in the IoT networks. Table 2 shows a key management technique for linking P (Figure 2) between two nodes in the same cluster and the same network. Link Q (Figure 2) is a communication link between two nodes $X_{1}$ and $X_{2}$ of separate clusters but of the same network, as depicted in Table 3. When two nodes $X_{3}$ and $X_{4}$ from different networks want to communicate with each other, they use connection R (Figure 2), shown in Table 4. Each link is described in the key management scheme in detail below.

### 7.1. The Key Formation Methods for Link P

We may potentially break down the key establishment method in the following steps:

***Pre-distribution state: the initial stage is the same for all sorts of connections***

According to a recent study, using a pre-distributed symmetric secret key to create secure channels across IoT devices is the most realistic choice. Before the deployment of IoT devices, the base station generates a key pool containing partial keys. The size of the key pool is an essential aspect to take into consideration. Since an encryption/decryption key should be 64 bits long and the key pool has 10,000 partial keys that are each 32 bits long, an IoT end device would need $\left\{\left(\frac{64}{2}\right)10,000\right\}$ bits, or 40 KB, to store all of the partial keys. The Wasp Motes has 128 K bytes of flash and 8 K bytes of SRAM. An IoT node can remove all the partial keys except those assigned to it after cluster formation, even though the proposed approach requires each IoT node to use up all the partial keys created at the base station.

So, if an IoT device has 1000 partial keys, it only needs 4 KB of storage space for the partial keys. When n partial keys are used, two nodes can be set up to $2n^{2}$ secret session keys. The number of session key candidates indicates the smallest number of session keys that could be made to encrypt or decrypt data. Before being put into use, each IoT end device is given a key pool of partial keys P, a single network key $N_{k}$, and a list of partial key index values L, with a unique identifier device MAC address. If a key pool has n number of partial keys, $\log_{2}n$ Bits are needed to uniquely identify each partial key, referred to as the index. An IoT node is first loaded with around 58 KB of data, which comprises 40 kilobytes for partial keys P, 17.5 kilobytes for the index list L, and 128 bits of the network key $N_{k}$ and identity MID. The construction of a cluster requires each node to allocate 4 KB of memory for partial keys, 1.25 KB of memory for the index list, and 128 bits of memory for the network key and identifier, resulting in a total memory requirement of 5.27 KB.

***The initial state of the network formation phase***

The unique identifier of each node and the single network key are used to securely distribute the administrative keys and verify the identity of each node, respectively. The suggested key management technique demonstrates each IoT device in a cluster during the initial step of setting up a network. The majority of IoT networks are deployed to protect or monitor vital facilities. In applications such as structural monitoring, it is reasonable to presume that the IoT field is only subject to intensive monitoring during the deployment phase, which does not typically continue for an extended period [43]. It is reasonable to presume that attackers are not actively capturing or attacking individual IoT nodes during this time. Therefore, it can be considered that a single network key is sufficient during the initial roll-out phase. An attacker may send powerful signals disguised as HELLO messages to persuade an IoT node to consider it a neighbour and so to join a cluster. As a result, after constructing a cluster, the proposed technique performs a three-tier verification check.

The cluster head receives all cluster members’ MIDs encrypted by the network key. After getting all of the MIDs, the cluster head sends them, along with its own MID and the cluster ID, to the base station for verification. The BS communicates with all cluster heads after validating the members of a cluster. The network key $N_{k}$ encodes all transmissions between the base station and the cluster heads.

For each cluster, the BS selects 1000 partial keys from the pool of keys. As part of the next step, each cluster will have a unique set of partial keys, which will be used to create communication keys. An index list of the nominated partial keys labelled as $LPK_{i}$ are created for each cluster i. The cluster head $CH_{i}$ receives the index list/partial key list $LPK_{i}$ from the base station. The $LPK_{i}$ is then distributed to all nodes of the cluster by the $CH_{i}$. When an IoT node has determined which partial keys it will use with its cluster head, it deletes the remaining partial keys from the key pool P.

***Communicating nodes and cluster head***

The IoT nodes are now prepared to set up communication keys. An IoT device node $X_{i}$ of cluster i only interacts with its cluster head $CH_{i}$ in the cluster structure. All nodes share the identical list of partial keys $LPK_{i}$ in cluster i. The cluster head $CH_{i}$ then delivers a unique order list $OCH_{i}$ to each member of cluster i, which includes the ordered list of index numbers of q partial keys chosen from $LPK_{i}$. $OX_{i}$ is generated by each member node $X_{i}$, which transmits it to $CH_{i}$ in a different order than $OCH_{i}$ but with the same cardinality as $OCH_{i}$’s order list. It’s now possible to create secret communication keys for each round between IoT node $X_{i}$ and cluster head $CH_{i}$. Concatenation is used to merge two partial keys into one communication key for convenience.

### 7.2. The Key Formation Methods for Link Q

The key management techniques for link Q are represented in Table 3. The following procedure can establish the secret keys between the two nodes $X_{1}$ and $X_{2}$ by employing link Q. The pre-distribution procedure and the step of interacting with the cluster leaders are the same for each specified link. The following is one way to describe the process of sharing the LPKs of both $X_{1}$ and $X_{2}$.

It is necessary for node $X_{1}$ and $X_{2}$ to exchange their respective lists of partial keys for them to be able to generate the communication keys. Through the cluster head $CH_{1}$, the node $X_{1}$ sends its preferred order list $O_{X_{1}}$ to the base station. The base station computed the partial key list of $X_{1}\left\{PLK\left(X_{1}\right)\right\}$, and then the list is transmitted to the node $X_{2}$ through the cluster head $CH_{2}$ in the network. Similarly, node $X_{2}$ communicates its order list $O_{X_{2}}$ to the node $X_{1}$ over $CH_{2}$, the base station, and $CH_{1}$.

### 7.3. The Key Formation Methods for Link R

Table 4 illustrates the key management strategies that should be considered for link R. By using link R, the following technique can be followed to determine the secret keys that are shared between the two nodes $X_{3}$ and $X_{4}$.

To generate the communication keys, the nodes $X_{3}$ and $X_{4}$ must exchange their generated specific key order list. Node $X_{3}$ transmits its preferred Order list $O_{X_{3}}$ to the base station by way of the cluster head $CH_{2}$. The order list $O_{X_{3}}$ is then sent from the base station to the node $X_{4}$ via the cluster head $CH_{3}$.

In the same way, node $X_{4}$ communicates its order list $O_{X_{4}}$ to node $X_{3}$ over $CH_{3}$, the base station, and $CH_{2}$.

## 8. Discussion and Evaluation

In the initial segment of this section, we provide a comprehensive security analysis and evaluation of the proposed key management scheme, illustrating its robustness against various attacks. Subsequently, we provide an evaluation of the proposed scheme.

***Security Analysis and Evaluation of the Proposed Key Management Scheme:***

In the event of the following attacks, the proposed method will be resistant.

*Secure from node capture:* An attacker can access any data on an IoT device if it is taken over and compromised [15] (partial keys, MID, network key $N_{k}$). However, an intruder cannot obtain the complete order list $O_{X_{i}}$ for nodes $X_{i}$ that are subject to frequent change. Since of this, even if a compromised device tries to connect with the cluster head or other devices inside or outside the network, it is impossible for them to do so because they do not have a complete order list.

*Eavesdropping resistance:* Typically, IoT devices connect via wireless networks. An attacker can easily capture messages when sent transparently across wireless networks [44]. Thus, an adversary could read the HELLO message between two neighbours during the key generation phase. However, he cannot compute the symmetric key since he is unaware of the partial keys $P_{K_{X_{i}}}$, the order list of partial keys $O_{X_{i}}$ and the network key $N_{k}$. Following the key establishment process, all communications between nodes are secured with the pre-distributed partial keys. As a result, an intruder could not see the contents of the communications being sent and received. So, the proposed system is strong enough to resist this attack.

*Forward and backward secrecy resistance:* Forward secrecy assurances that a node cannot decrypt fresh messages with an old key. Backward secrecy ensures a node cannot use a new key to decrypt old messages. Node capture attempts can be avoided using forward and backward secrecy [45]. In the proposed system, when a node is compromised or departs the network, its neighbours erase the order list $O_{X_{i}}$ they shared with it from their memories and update it. As a result, the leaving node cannot use the CA and order list to create a new connection with its neighbours, as these nodes have already erased this CA from their memories. In addition, because they are unable to access constantly updated order lists from other nodes or the cluster head, these neighbours cannot establish new connections with the departing node. Since it no longer has access to the order list of partial keys, a node that has left the network cannot decrypt new messages using the partial keys. Similarly, when a node rejoins the network, it cannot decode earlier messages since the current order list is different from the earlier ones. Therefore, this plan provides both forward and backward secrecy.

*Sybil attack resistance:* A single malicious device gives several identities to other nodes during a Sybil attack. Each node’s identification must be verified to be safe against such an attack [46]. A cluster head or another reliable entity can do this task directly. The CH is what controls the Internet of Things nodes that belong to a specific group. As a result, CH is deemed to be the certificate authority (CA) for the same set of Internet of Things nodes that are included in the group of devices, comparable to the distribution of implicit certificates. During the phase before distribution, it is assumed that edge nodes will be familiar with the CH. Therefore, our system is resistant to attacks of this nature.

*Replay attack resistance:* As the name suggests, a replay attack involves eavesdropping on other devices’ communications and exploiting that information later to compromise security [47]. Our approach incorporates random and unpredictable numbers, such as a random order list, to prevent a replay attack. Additionally, because the order list routinely refreshes keys, an attacker cannot use the messages of one old session in another new session. Suppose an attacker passively eavesdrops and obtains the refresh message sent by a cluster head $CH_{i}$ to its resource-constrained nodes during the key refresh phase. In that case, the intruder will not be able to decrypt that message because he does not know the order list $O_{X_{i}}$ that leads to the new key. In fact, the node $X_{i}$ has already received a new message from the close cluster head $CH_{i}$ or another one, so it has already updated its order lists to update the secret keys and erase the old ones from its memory. Consequently, a replay attack can be detected by the receiver node.

*Secure from man-in-the-middle attacks:* Man-in-middle attacks are where the attacker is placed between two entities communicating, allowing them to read and/or alter the communications being exchanged. At this point, neither party has any idea that a third party is involved [48,49]. IoT devices and the cluster head know that the attacker is there because they get this unauthorised user’s messages. Also, only HELLO messages are not encrypted, but they include the MID and network key $N_{k}$ so that the recipient can tell if the messages were changed while they were being sent. The secret keys encrypt all other communications transmitted in our method. This makes it difficult for an attacker to read or alter the communications. In other words, the attacker cannot compromise any data’s accuracy, completeness, or confidentiality. Our plan is, therefore, resistant to man-in-the-middle attacks.

***Evaluation of the proposed scheme:***

The proposed technique is evaluated using a variety of metrics, such as (a) aggregation, (b) time complexity, (c) memory requirement, (d) power management, (e) resilience, (f) the number of session key candidates, (g) scalability, (h) key connectivity, (i) the complexity of storage, (j) the complexity of processing, (k) the complexity of communication, and (l) revocation.

Once a communication technique is set up, the number of messages required to implement security measures must be considered before any other considerations are made. Node readings from multiple nodes can be processed at one of many aggregation points, which cuts down on the number and size of messages sent and saves energy. Device readings from nearby nodes are gathered by an aggregation point and transmitted in a single message. Cluster heads $CH_{i}$ can serve as aggregation points in a cluster-based structure.

There is only one message sent to the cluster head by each member node and only one message received by the cluster head in the proposed key management technique for establishing one hundred secret communications keys. The total number of messages sent and received by i number of nodes in a cluster equal 2(i−1). So, devices do not have to exchange a secret key every time they want to communicate with each other.

In the proposed key management scheme, when two nodes want to establish encryption/decryption keys, each node transmits a message to its peer node containing an order list of its preferred partial keys. Notably, these messages do not carry the actual partial keys; rather, they encompass the indices of corresponding partial keys. The indices are arranged in the sequence that must be utilised to construct full keys.

Traditionally, when two nodes seek to establish a complete key, they engage in message exchange to establish cryptographic keys. Each node sends a message, containing information related to partial keys, to its peer node. Thus, at least two message exchange is required to establish a full key. In contrast, the proposed technique can generate multiple full keys utilising two message exchanges. For example, assuming the order list comprises indices corresponding to 20 partial keys, 20 full keys can be generated with just 2 message exchanges. Given that message transmission constitutes the most time-intensive aspect of the key establishment, the proposed protocol significantly diminishes the overall time complexity.

**Figure 3:**
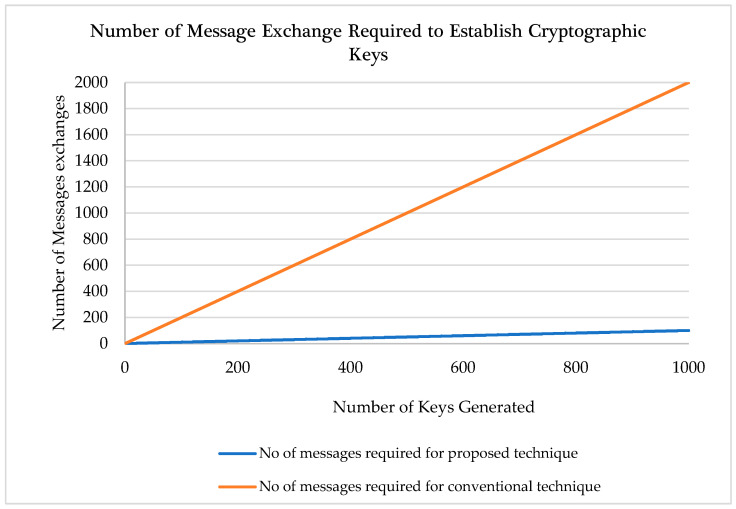
Relationship between the number of message exchanges and the number of keys generated. The relationship demonstrates the time complexity of the proposed technique.

The graphical representation (Figure 3) illustrates a direct correlation between time complexity and the quantity of exchanged messages: a more significant number of messages corresponds to increased time consumption. For instance, the proposed technique requires 50 message exchanges to establish 500 full keys, whereas the conventional technique requires 1000 message exchanges, as shown in Figure 3.

IoT devices usually have limited resources, so presented lightweight key management protocols are a good fit. The key length used in Table 5 is the length of a partial key (or half key) rather than a full key used by our cryptographic algorithm to encrypt or decrypt a message. Our algorithm combines two of these partial keys to form a full key, leading to a total key size of 64 bits. Moreover, it should be noted that each communication round employs fresh partial keys. Key freshness means no pair of partial keys are utilised more than once in any communication round. Using partial keys instead of full keys provides added benefits, especially for resource-constrained IoT devices such as:

Lower Storage Needs: IoT devices typically come with constrained storage capacities. Using partial keys can substantially cut down the storage needs for storing keys. This method thus promotes a more resource-efficient usage in such limited-capacity IoT devices.

Enhanced Security: Partial keys provide an additional layer of security by reducing the exposure of the full key. Even if a partial key is compromised, it would be much more challenging for an attacker to reconstruct the full key. This helps to mitigate the risk of unauthorised access to sensitive information.

Improved Key Management: Managing and distributing full keys in a large-scale IoT deployment can be challenging. By using partial keys, key management becomes more manageable and scalable. It simplifies the process of distributing keys to devices and managing their lifecycle.

Increased Efficiency: Cryptographic operations, such as encryption and decryption, can be computationally intensive for resource-constrained IoT devices. Using partial keys can reduce the complexity of these operations, resulting in improved efficiency and faster processing times.

Flexible Access Control: Partial keys can be used to implement access control mechanisms in IoT systems. Different levels of access can be granted based on the possession of specific partial keys. This allows for fine-grained control over data access and enhances overall security.

Reduced Consequences of Key Breach: If a partial key happens to be compromised, the repercussion is restricted to a portion of the whole key. This narrows down the extent of potential harm and allows for more rapid recovery procedures.

Table 5 demonstrates the memory needs of IoT nodes using the suggested techniques for varying numbers of secret keys. In addition to that, we employed a pre-distribution method for the partial keys. Pre-distribution is better for devices with limited resources, such as IoT devices, according to research, because it reduces the number of communications needed to distribute the keys of the pools [24,50]. The calculation shows that about 5 KB of storage is required to hold the index list, network ID, and 1000 partial keys.

**Figure 4:**
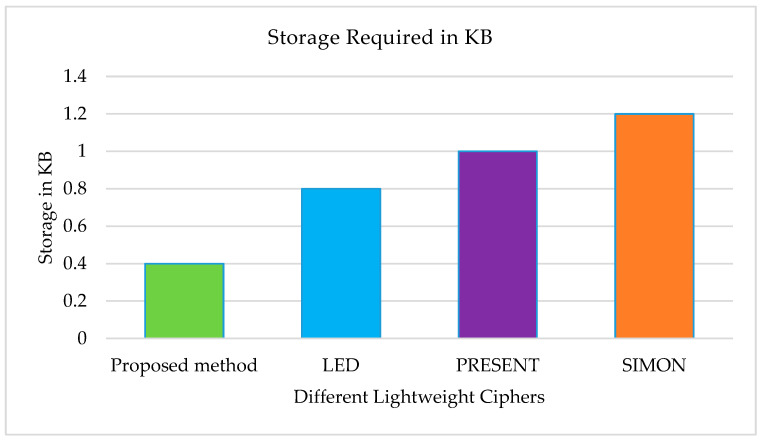
Storage requirements by different lightweight block ciphers.

In the proposed approach, each node stores 32-bit partial keys rather than complete keys. Combining two partial keys generates a full key of 64 bits. Therefore, to generate 100 full keys, each node must store partial keys that consume a mere 0.40 KB of space. In contrast, lightweight block ciphers such as LED, PRESENT, and SIMON require 0.80 KB, 1 KB, and 1.2 KB of storage to generate the same number of full keys [19]. Figure 4 demonstrates that the proposed technique requires less space than these widely recognised lightweight block ciphers.

When a network is resilient, it can continue functioning securely even after several security breaches. Resilience can be accomplished in one of three ways: (a) the likelihood that a link is broken when an attacker captures a device; (b) A node’s security credentials are obtained when it is taken over by an attacker; or (c) the number of sensor nodes must be taken over for a whole network to be broken [51]. An intruder who captures an IoT end device cannot obtain full encryption and decryption keys. Using partial keys rather than full ones makes the system more secure. For example, a hacker can attack a sensor node physically and read secret information from the device’s memory. The compromised device has a copy of the previously utilised order list, but it cannot be used to communicate further. Furthermore, the capturing node cannot acquire a new order list, indicating that even if an intrusion occurs, a partial key cannot be used to construct a full key for communication. This technique is to make sure that the order list is always up to date.

**Figure 5:**
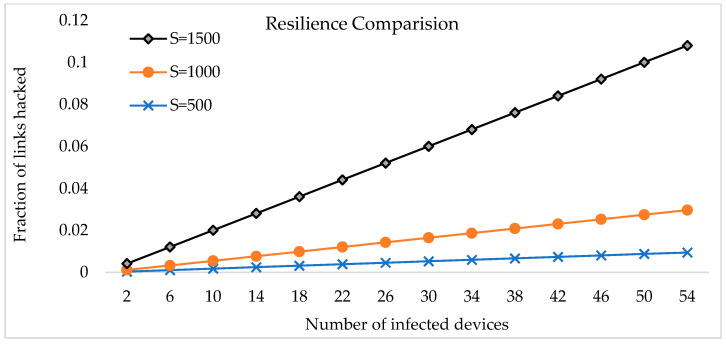
The resilience of the suggested key management system when each node contains 20 partial keys.

We figure out how resistant a technique is to node capture by figuring out what percentage of all network communications are affected by the capture of n nodes. Exclude the communications in which the captured devices are directly involved. We examine how our method improves the security of an IoT network in the face of a node capture threat. We achieve this by determining how many links in the network a hacker can listen to (indirectly) by obtaining keys from captured devices. X and Y, for example, are two IoT network devices the attacker fails to capture. Using a subset of the key pool, it is possible to check how likely an attacker can decrypt their secret keys. With m partial keys, the number of full keys can be found by using $m_{P_{2}}$, where P is the permutation function. The n number of devices is captured from the total number of nodes S. As each device can hold m partial keys, the probability that the security has not been broken can be written as $P\left(not_{compromised}\right)={\left(1-n\times \frac{m_{P_{2}}}{s_{P_{2}}}\right)}^{n}$. Since n nodes have been taken, the likelihood that any secure link established during the key setup phase between two uncompromised nodes is compromised is $1-{\left(1-\frac{m_{P_{2}}}{s_{P_{2}}}\right)}^{n}$. Based on the number of nodes the attacker controls, you can see in Figure 5 how this dynamic evolves because the *x*-axis represents the overall number of infected nodes, no matter how large the network is in total. The *y*-axis, however, shows what percentage of all network communications were compromised.

**Figure 6:**
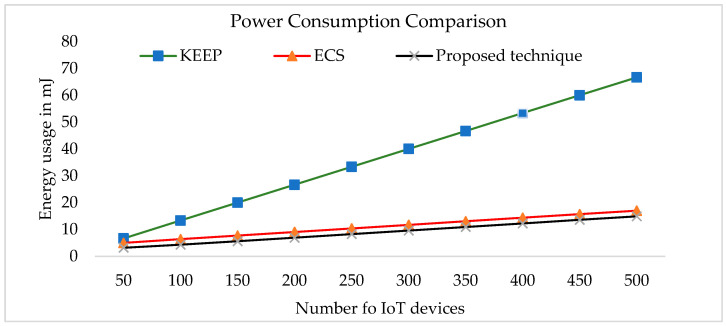
Comparison of the proposed technic’s power consumption.

IoT network device power control is critical. At full power, the batteries in the Berkeley Mica Mote last about two weeks. For a sensor network to last for years, it must run at a duty cycle of 1% or less. Since the radio uses three times as much power when transmitting than receiving, it is best to leave it in sleep mode most of the time. Each bit transferred needs as much energy as 800–1000 instructions; hence, message expansion induced by security methods is expensive. Instead of sending and receiving partial keys in each round, the future secret keys are calculated by exchanging the order list with the cluster head. Instead of sending and receiving partial keys in each round, we calculate upcoming secret keys by gathering the order list $O_{X_{i}}$ from a cluster head CH or device X. We determined how much energy the IoT nodes utilised to set up the secret keys for transmission and compared it to the energy used by the recently developed Elliptic Curves Signcryption (ECS) and Key Exchange and Encryption Protocol (KEEP) [52,53]. Figure 6 compares the three key management strategies. The proposed approach sends fewer messages per band than ECS and KEEP. The sensor nodes in the proposed system do not have to send any further messages for the next hundred data collection rounds once they have exchanged the index file of the nominated partial key list.

Session key candidates indicate how many keys are needed to encrypt or decode a given bit of information. If an intruder knows the list of partial keys, they cannot get a secret key since this session key domain grows exponentially with the number of partial keys given to the end device. Session key domains grow exponentially with the number of partial keys supplied to a device, as shown in Figure 5. This means that even if an attacker knows the $LPK_{i}$, it is extremely difficult to discover a secret key.

**Figure 7:**
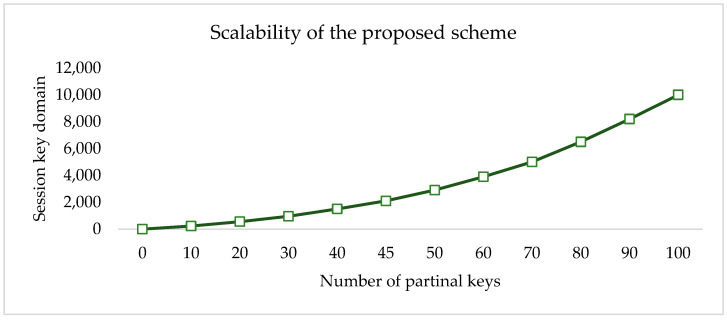
Number of session key candidates.

Scalability, or the capacity to manage larger networks, is the most important quality for an IoT network. IoT end devices must have enough storage space to store the security credentials required to enable larger networks. According to Figure 7, the proposed method is highly scalable. Each end device only needs to store 1000 partial keys, which takes up about 5 KB from the half a million session keys. This capacity shows that the network can support a large number of devices.

Pre-distribution techniques for random keys are incomplete without considering the importance of key connectivity. This study examines whether two or more Internet of Things devices will keep the same key or keying information to build up keys for a pair, group, or network. Using this proposed method, all group members have access to the same LPK or partial key set. As a result of the sharing, this technique is always connected.

The number and size of packets transmitted and received by an IoT device evaluate its communication complexity. Node communication consumes the most power of all IoT functions. A device connects with other devices using only the order list instead of partial keys in our proposed method. As a result, packet sizes are kept to a minimum. The degree of processing complexity is determined by the number of unit functions run during the process. Searching for keys on a keychain and other operations like pseudo-randomness, hashing, message authentication codes, XOR, and so on are all examples of unit operations. VecMul (size) multiplies two vectors of specified sizes, while PolyEval evaluates a polynomial at a specified point. The concatenation function is used in the proposed solution, eliminating the need for complex operations.

The process by which the system examines an IoT device’s validity to determine if the device is malicious or compromised is known as key revocation. Key revocation aims to eliminate any potentially harmful hardware from the network. If a node is suspicious, the revocation’s attribute determines whether it can be removed from the system dynamically. When an attacker obtains pre-distributed keying information, key revocation and rekeying become necessary. Malicious nodes can be removed from the network without a BS by using a distributed node revocation mechanism. The base station’s revocation methods may also slow down the deletion of a node because of the high latency between the nodes and the base station. Revocations necessitate quick responses since a detected attack must be isolated from the network before it can accomplish any severe damage. We provide a node revocation technique for this protocol to minimise the limitations of a base station-dependent revocation protocol.

In this scenario, we assume that each IoT node has a mechanism to identify if its neighbouring nodes are infected. This system’s encryption/decryption keys are generated randomly from the order list, and each node is assigned a unique identification number throughout each cycle. A node can merge the unique identification number while sending information to the next node. A node uses a new session key and a unique identification number to identify a fake device. The cluster head can be addressed if a device receives encrypted data with a key that does not match its secret key. The cluster head would ask the node’s predecessor if they noticed anything unusual about the session keys. To create a new chain, if the local-level leader suspects it is an adversary, it alerts everyone else in the chain. These features make it more difficult for a malicious attacker to attack an IoT device.

In our tailored approach designed for IoT applications, our primary strategy revolves around utilising symmetric cryptography to manage both the encryption and decryption processes. Symmetric cryptography demonstrates remarkable efficiency, particularly suited for resource-constrained devices, as it hinges upon a shared key between the sender and receiver for data protection. Nevertheless, as we aspire to advance and fortify the security dimensions of our methodology, our forthcoming endeavours will delve into the incorporation of asymmetric cryptography keys. This transition holds the potential to usher in an elevated tier of security and authentication, addressing specific inherent challenges associated with symmetric cryptography.

## 9. Conclusions

This research proposed a secure key management strategy for resource-constrained environments based on the pre-distribution of partial keys. This strategy is implemented in a cluster-based IoT network, which is a prevalent scenario in the Internet of Things. The proposed approach involves the generation of symmetric pairwise secret keys between each IoT node and the cluster head, as determined by the order of the pre-distributed list. This research indicates that the proposed technique is more efficient than existing random key pre-distribution protocols in terms of space consumption, communication overhead, and the number of session key candidates. Additionally, the system has been shown to be secure against various attacks, as the keys are frequently refreshed without being transmitted between devices. The findings of this study highlight the efficiency and scalability of the proposed key management strategy. Future research will focus on developing a lightweight block cipher technique using this key management method to provide secure and efficient communication in resource-constrained IoT environments.

## Figures and Tables

**Table 1:** The suggested key management strategy’s notation.

Notation	Description
n	Number of clusters in total
$C_{i}$	The i-th cluster
$A_{i}$	An IoT node that belongs to the cluster head $C_{i}$
$CH_{i}$	Head of the cluster $C_{i}$
N	IoT devices deployed in total
BS	The base station
k	Total number of partial keys
E	Encryption feature
P	A collection of partial keys called a key pool
$PK_{i}$	i-th partial key
$N_{k}$	Network key
$idx_{n}$	Index of n-th partial key
L	Index list of partial keys
$L_{i}$	List of partial key index entries for cluster $C_{i}$
$LPK_{i}$	List of partial keys/index list nominated for cluster $C_{i}$
MID(x)	Media access control (MAC) address specific to device x
$O_{X_{i}}$	A set of orders of partial keys selected by the IoT node $X_{i}$
∥	The concatenation operation
$R_{X_{i}}^{q}\left(L_{i}\right)$	The function used by the node $X_{i}$ to randomly select q number of indexes from $L_{i}$
X→Y	A message is sent from node X to node Y
$K_{XY}^{t}$	For the t-th round, a secret key is established between IoT nodes X and Y
$E_{N_{k}}\left(M\right)$	Network key $N_{k}$ is being used to encrypt message M

**Table 2:** Proposed key management scheme.

***A. Pre-distribution state: the initial stage is the same for all sorts of connections***
Each IoT device is pre-loaded with the following partial keys prior to deployment: (a) The base station generates a set of partial keys, $P=\left\{PK_{1},\;PK_{2},\;\ldots \ldots ,\;PK_{k}\right\}$; (b) An index list $L=\left\{idx_{1},\;idx_{2},\;\ldots \ldots ,\;idx_{K}\right\}$ containing an identity for each key in key pool P, in this manner $L\left(idx_{n}\right)=PK_{n}$ for n=1, 2, 3, ……, k; (c) A network key $N_{k}.$; (d) MAC address a unique identifier for IoT node $MID_{i}$ here i=1, 2, 3, ……, N.
***B. Initial state of network formation phase***
(a) Members of the cluster $C_{i}$, as (i=1, 2, 3, ……, n), send encrypted MIDs to the cluster head $CH_{i}:X_{i}\;\to CH_{i}\;:E_{N_{k}}\;\left\{MID\left(X_{i}\right)\right\}$, here, $X_{i}\;\in C_{i}$, after it has established the connection; (b) For verification, the cluster head $CH_{i}$ gathers all the MAC addresses and transmits them to the BS along with its own MAC address: $CH_{i}\;\to BS:E_{N_{Kk}}\text{ \{}MID_{i}\left(X_{i}\right)\;|\;X_{i}\;\in C_{i},\;MAC_{i}\left(CH_{i}\right)\}$; (c) A list of partial keys $LPK_{i}$ from the key pool P is selected for each cluster and sent to the cluster head of a cluster with an index $L_{i}\;\left(L_{i}\subset L\right)$, if authentication is successful: $C_{i}:BS\to CH_{i}:E_{N_{k}}\text{\{}L_{i}\;|\;\forall \left(L_{i}idx_{x}\;\in LPK_{i}\right)\}$; (d) All cluster members receive an encrypted list of indexes from $CH_{i}$: $CH_{i}\to \text{\{}X_{i}\;|\;X_{i}\in C_{i}\}:\;E_{N_{Kk}}\left(L_{i}\right);$ (e) In order to determine IoT node’s own partial key list, each node in the cluster deletes the partial keys that have not been mapped by $LPK_{i}$ from key pool: $LPK_{i}=\{L\left(idx_{x}\right)|idx_{x}\;\notin L_{i}\}$.
***C. Communicating nodes and cluster head in the same cluster***
Members $X_{i}$ and cluster head $CH_{i}$ of a cluster establish an encryption/decryption key by interacting with each other: (a) $CH_{i}\to X_{i}:E_{N_{k}}\left(O_{CH_{i}}\right)=E_{N_{k}}\;\left\{R_{CH_{i}}^{q}\left(L_{i}\right)\right\}$ $X_{i}\to CH_{i}:E_{N_{k}}\left(OX_{i}\right)=E_{N_{k}}\{R_{X_{i}}^{q}\left(L_{i}\right)\}$, $X_{i}$ and $O(CL_{i})$ have the same cardinality, which we can denote as q; (b) The secret key for the *t*-th round is computed as follows by nodes $X_{i}$ and $CH_{i}$: $K_{X_{i}CH_{i}}^{t}\left\{L_{i}\left(O_{X_{i}}\left[t\right]\right)∥L\left(O_{CH_{i}}\left[t\right]\right)\right\}$, where $O_{X_{i}}\left[t\right]$ returns the $O_{X_{i}}$’s t-th index. $X_{i}$ and $CL_{i}$ figure out the secret key for the next round as follows $K_{X_{i}CH_{i}}^{t+1}=\{L_{i}(O_{X_{i}}\left[t+1\right]\}∥L\left(O_{CH_{i}}\left[t+1\right]\right)$} and so on. $K^{t}A_{i}CH_{i}(L_{i}(O_{A_{i}}\left[t\right]\;||\;L\left(O_{CH_{i}}\;\left[t\right]\right))$ where $O_{A_{i}}\;\left[t\right]$ returns the t-th index of $O_{A_{i}}$ $O_{A_{i}}\left[t\right]$ returns $O_{A_{i}}$’s t-th index. For the next round, $A_{i}$ and $CL_{i}$ compute the secret key as $K^{t+1}A_{i}CH_{i}=(L_{i}(O_{A_{i}}\left[t+1\right]\;||\;L\left(O_{CH_{i}}\;\left[t+1\right]\right))$ and so forth.

**Table 3:** Two IoT nodes are in the same network but in separate groups (Link Q).

***A. Transmitting the order list*** $O_{X_{1}}$ ***from*** $X_{1}$ ***to*** $X_{2}$ ***and vice versa***
(a) Sending a list of orders to $CH_{i}$ from node $X_{1}$ $X_{1}\to CH_{1}:E_{N_{k}}\left(O_{X_{1}}\right)$; (b) The order list is sent to base station by node $CH_{1}$ $CH_{1}\to BS:E_{N_{k}}\left(O_{X_{1}}\right)$; (c) A copy of the $O_{X_{1}}$ order list is forwarded from BS to $CH_{2}$ $BS\to CH_{2}:E_{N_{k}}\left(O_{X_{1}}\right)$; (d) $CH_{2}$ transmits the list $O_{X_{1}}$ to $X_{2}$ in $CH_{2}$ $CH_{2}\to X_{2}:E_{N_{k}}\left(O_{X_{1}}\right)$. Similar to $X_{1}$, $X_{2}$ transmitted back to $X_{1}$ its order list $O_{X_{2}}$ (a) Transmit an order list to $CH_{2}$ from node $X_{2}$ $X_{2}\to CH_{2}:E_{N_{k}}\left(O_{X_{2}}\right)$; (b) The order list is sent to base station by node $CH_{2}$ $CH_{2}\to BS:E_{N_{k}}\left(O_{X_{2}}\right)$; (c) $CH_{1}$ receives the order list $O_{X_{2}}$ list from base station $BS\to CH_{1}:E_{N_{k}}\left(O_{X_{2}}\right)$; (d) $CH_{1}$ transmit the list $O_{X_{2}}$ to $X_{1}$ $CH_{1}\to X_{1}:E_{N_{k}}\left(O_{X_{2}}\right)$.
***B. Communicating nodes*** $X_{1}$ ***and*** $X_{2}$
The nodes act as follows to establish an encryption/decryption key between $X_{1}$ and $X_{2}$. The secret key for the t-th round is now computed by both end devices $X_{1}$ and $X_{2}$: $K_{X_{1}X_{2}}^{t}=LPK\left(X_{1}\right)\left[t\right]∥LPK\left(X_{2}\right)\left[t\right]$. The secret key is computed as follows for the following round by $X_{1}$ and $X_{2}$: $K_{X_{1}X_{2}}^{t+1}=LPK\left(X_{1}\right)\left[t+1\right]∥LPK\left(X_{2}\right)\left[t+1\right]$.

**Table 4:** Two IoT nodes are distributed over two clusters and two networks (link R).

***A. Transmitting the order list of*** $O_{X_{3}}$ ***from*** $X_{3}$ ***to*** $X_{4}$ ***and vice versa***
(e) Sending a list of orders to $CH_{2}$ from node $X_{3}$, $\;X_{3}\to CH_{2}:E_{N_{k}}\left(O_{X_{3}}\right)$; (f) The order list is sent to the base station by node $CH_{2}$, $\;CH_{2}\to BS:E_{N_{k}}\left(O_{X_{3}}\right)$; (g) A copy of the $O_{X_{3}}$ list is forwarded from BS to $CH_{3}$, $BS\to CH_{3}:E_{N_{k}}\left(O_{X_{3}}\right)$; (h) $CH_{3}$ transmits the list to $X_{4}$, $\;CH_{3}\to X_{4}:E_{N_{k}}\left(O_{X_{3}}\right)$. Similar to $X_{3}$, $X_{4}$ transmitted back to $X_{3}$ its order list $O_{X_{4}}$ (e) Transmit an order list to $CH_{3}$ from node $X_{4}$, $\;X_{4}\to CH_{3}:E_{N_{k}}\left(O_{X_{4}}\right)$; (f) The order list is sent to the base station by node $CH_{3}$, $CH_{3}\to BS:E_{N_{k}}\left(O_{X_{4}}\right)$; (g) $CH_{2}$ receives the $O_{X_{4}}$ list from the base station. $BS\to CH_{2}:E_{N_{k}}\left(O_{X_{4}}\right)$; (h) $CH_{2}$ transmit $O_{X_{4}}$ to $X_{3}$, $\;CH_{2}\to X_{3}:E_{N_{k}}\left(O_{X_{4}}\right)$.
***B. Communicating nodes*** $X_{3}$ ***and*** $X_{4}$
The nodes act as follows to establish an encryption/decryption key between $X_{3}$ and $X_{4}$. The secret key for the t-th round is now computed by both end devices $X_{3}$ and $X_{4}$: $K_{X_{3}X_{4}}^{t}=LPK\left(X_{3}\right)\left[t\right]∥LPK\left(X_{4}\right)\left[t\right]$. The secret key is computed as follows for the following round by $X_{3}$ and $X_{4}$: $K_{X_{3}X_{4}}^{t+1}=LPK\left(X_{3}\right)\left[t+1\right]∥LPK\left(X_{4}\right)\left[t+1\right]$.

**Table 5:** Storage is needed for a different number of partial keys.

Partial Key Bits	No of Partial Keys	Index List	Network Key	Total in KB
32	50	350	64	0.25
32	100	700	64	0.48
32	250	2000	64	1.23
32	500	4500	64	2.51
32	1000	10,000	64	5.13
32	5000	65,000	64	27.47
32	10,000	140,000	64	56.16
32	20,000	300,000	64	114.75

## Data Availability

Not appliable.
